# Supplementary material for: Conjunctive encoding of exploratory intentions and spatial information in the hippocampus
Source: Nat Commun. 2024 Apr 15;15:3221. doi: 10.1038/s41467-024-47570-4 (PMC11018604; doi:10.1038/s41467-024-47570-4)
Supplement: Supplementary file 1 — Supplementary Information [file 41467_2024_47570_MOESM1_ESM.pdf]

Supplementary Information

Conjunctive encoding of exploratory intentions and spatial information in the hippocampus

Yi-Fan Zeng, Ke-Xin Yang, Yilong Cui, Xiao-Na Zhu, Rui Li, Hanqing Zhang,

Dong Chuan Wu, Raymond C. Stevens, Ji Hu & Ning Zhou

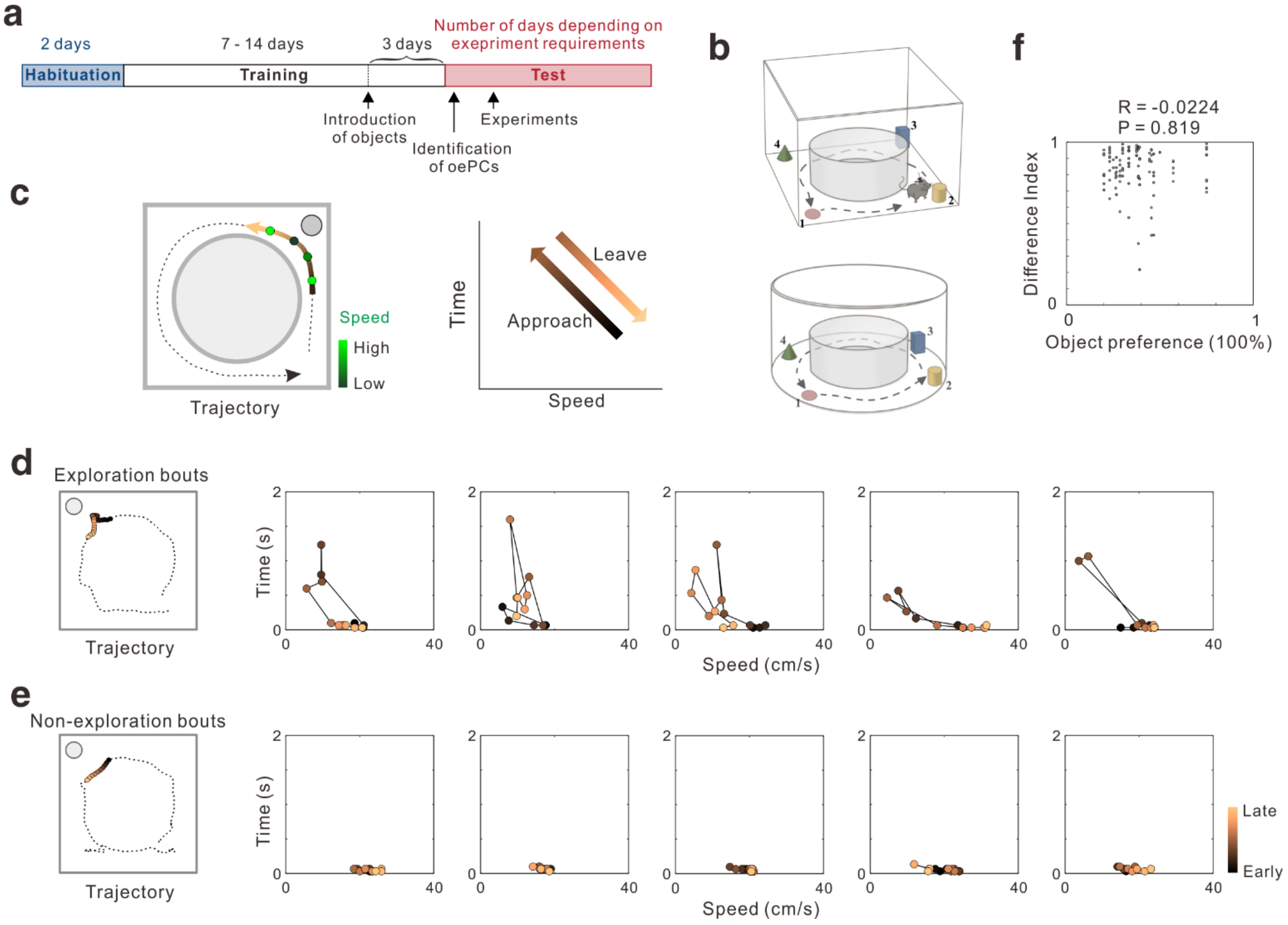

Supplementary Figure 1. Experimental timeline and behavioral variable analysis. (**a**) Schematic depiction of the experimental timeline. The mice were habituated to the apparatuses for two days, and trained for 7-14 days. In the last three days of training period, objects were introduced at the fixed locations of the maze. (**b**) Diagrams illustrating the object and reward locations in the square and circular mazes. Unless otherwise specified, rewards were consistently administered at location 1, while objects were positioned at positions 2-4. (**c**) Left: The mouse showed distinct behaviors during exploration bouts, such as reducing running speed when approaching the targeted object, sometimes followed by a near-complete stop. After exploration, the animal left the object with increasing speed, and the time spent near the object was also increased during exploration. In contrast, during non-exploration bouts, the running speed and time spent at each location along the trajectory were relatively constant. Right: Hypothetical relationship between speed and time along the trajectory during exploration behaviors, with color-coding to illustrate the corresponding locations in the left panel. (**d**) Example laps showing behavioral parameters during exploration, including the trajectory of the mouse in temporal sequence during an exploration bout (with 33.3 ms interval between every two positions) and the remaining part of the lap (dashed trace). The speed-time relationship at each time point according to temporal sequence is shown on the right. (**e**) The same as in (d) but for non-exploration behaviors. Note that the speed-time plots are clearly different between exploration and non-exploration behaviors. (**f**) No significant correlation was observed between the percentage of exploratory laps relative to the total laps, reflecting animals’ object preference, and the difference index values.

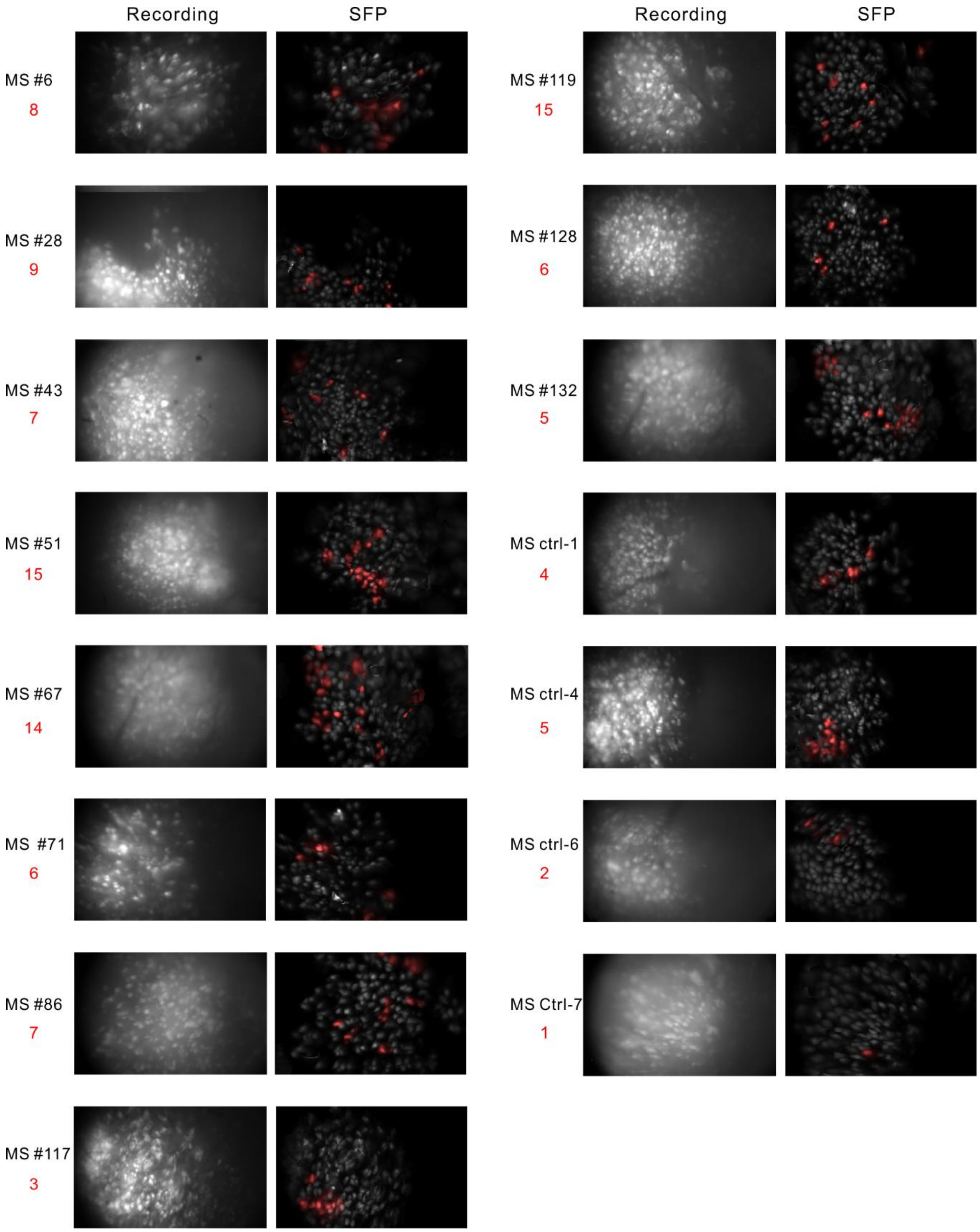

Supplementary Figure 2. Maps of object exploration-dependent place cells (oePCs). Images showing maximum projection from original Ca^{2+} recording frames (left) and the distribution of oePCs (red) in spatial footprint (SFP) (right) in 15 mice. Numbers in red indicate the number of oePCs.

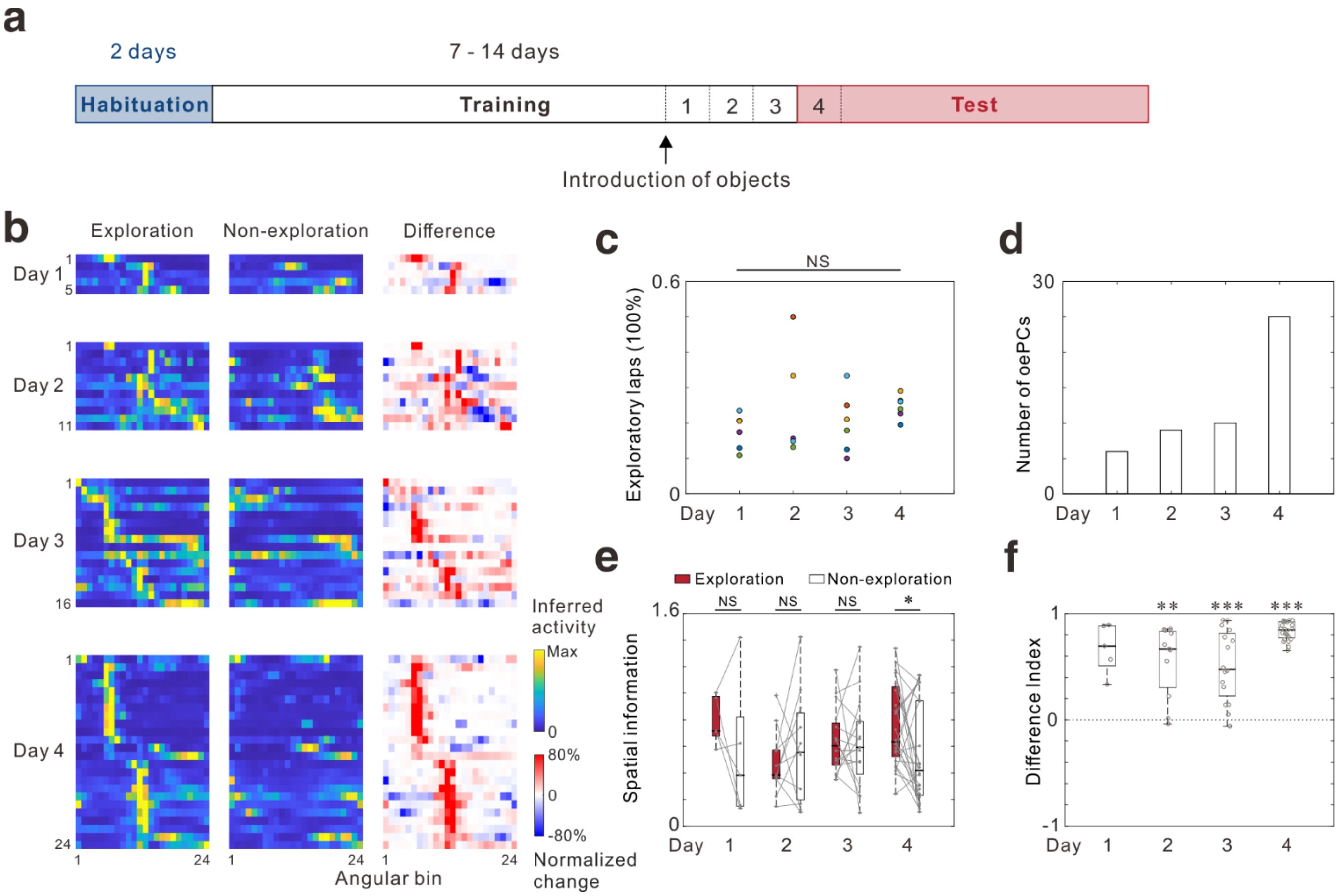

Supplementary Figure 3. The emergence of object exploration-dependent place cells (oePCs). (**a**) Schematic depiction of the experimental timeline. Ca^{2+} imaging was performed daily from Day 1 to Day 4. (**b**) The oePCs were identified from the recording sessions of Day 4, sorted by place field centers. The oePCs in Day 4 were tracked in Day 1-3, and the consistently active cells were shown for the lap-average activity in exploration and non-exploration laps. Inferred neuronal activity were normalized to the peak firing rate of each neuron and sorted according to angular position. The right panels show difference between the activity map in exploration and non-exploration laps. (**c**) The percentage of exploratory laps relative to total laps did not show significant difference across Day1 to 4. Dots with the same color indicate the same mouse (n = 5 mice). Statistical significance was determined by Kruskal-Wallis H tests. (**d**) The numbers of cells that met the criteria for being an oePC in each day. (**e**) Spatial information between exploration (red) and non-exploration laps (white) in oePCs shows statistical significance in Day 4 (determined by paired Wilcoxon signed rank tests). (**f**) Difference index in oePCs in Day 1 to 4. The median values of difference index are significantly higher than zero in Day 2, 3, and 4, as determined by Wilcoxon signed rank tests. Box plots show the median (horizontal line), 25–75% range (box) and outliers (whiskers). *** for $P < 0.001$, ** for $P < 0.01$, and * for $P < 0.05$, and NS for no significance. Data and statistical analyses are reported in the Source Data file.

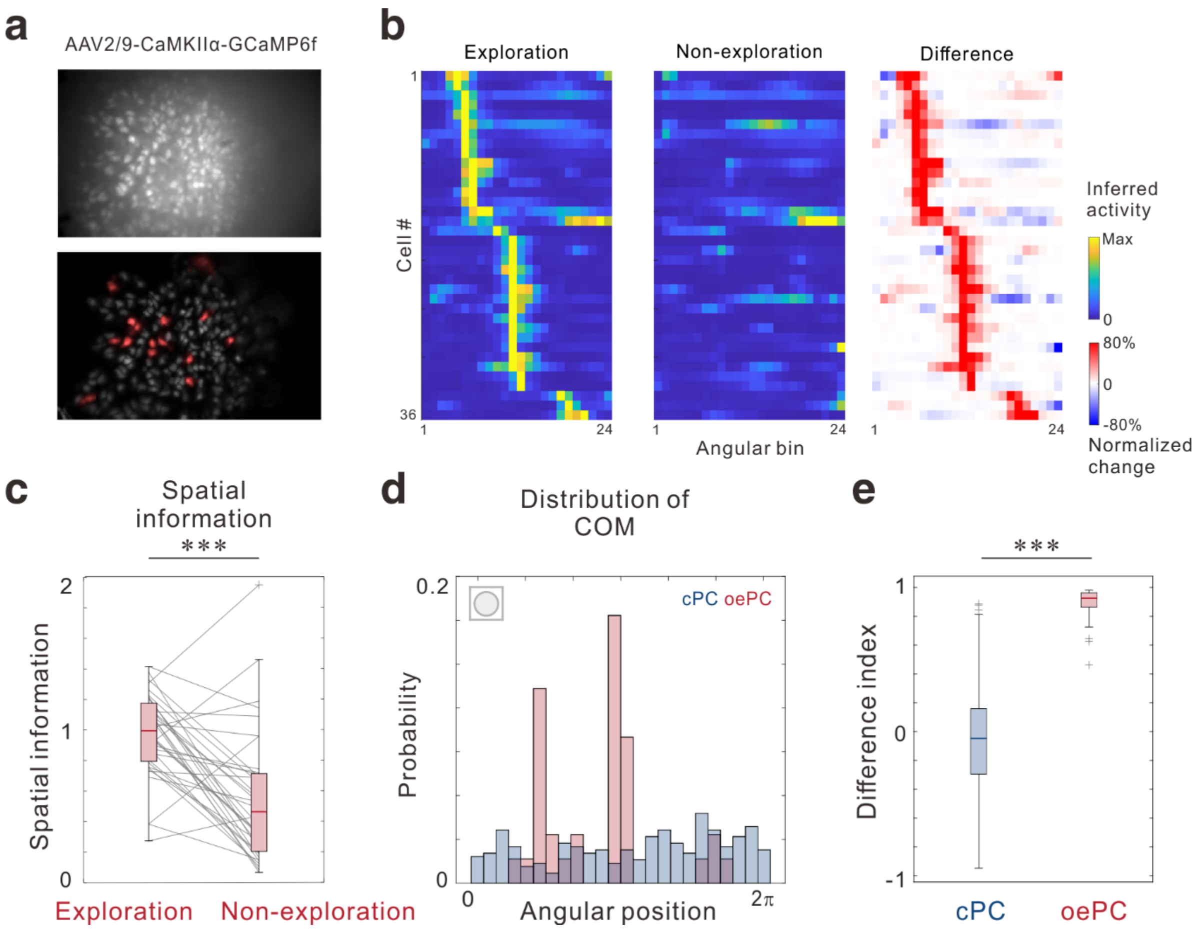

Supplementary Figure 4. Identification of object exploration-dependent place cells (oePCs) in excitatory principal neurons. (**a**) Images showing a representative experiment in CaMKIIα-GCaMP6f-expressing CA1 neurons, including the maximum projection from original Ca^{2+} recording frames (top) and the distribution of oePCs (red) in SFP (bottom). (**b**) Lap-average activity of oePCs in exploration and non-exploration laps across three mice, sorted by place field centers. Neuronal activity was normalized to the maximum activity of each neuron and sorted according to angular position. The right panel shows difference between the activity map in exploration and non-exploration laps. (**c**) Difference in spatial information between exploration and non-exploration laps in oePCs. $P = 3.36 \times 10^{-5}$; paired Wilcoxon signed rank test. (**d**) distribution of COM in cPCs (blue, n = 263) and oePCs (red, n = 36 in 3 mice). (**e**) Difference index was different in cPCs and oePCs. $P = 9.39 \times 10^{-22}$; two-sided Mann-Whitney U test. Box plots show the median (horizontal line), 25–75% range (box) and outliers (whiskers). *** for $P < 0.001$, ** for $P < 0.01$, and * for $P < 0.05$, and NS for no significance. Data and statistical analyses are reported in the Source Data file.

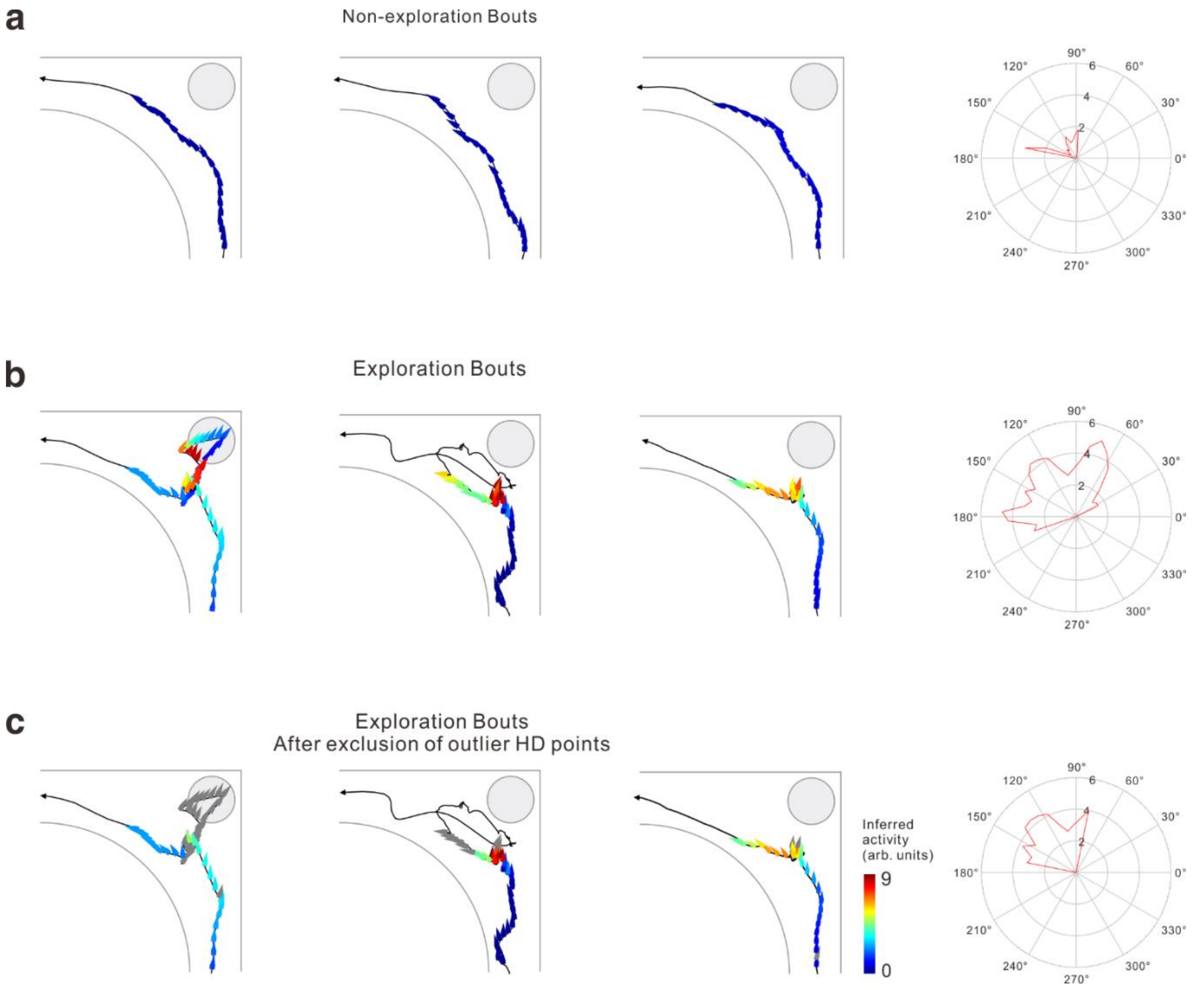

Supplementary Figure 5. Activity maps in relation to head directions. The activity maps illustrate the inferred activity of a representative oePC in relation to head direction (indicated by arrows) at various time points along the moving trajectory during three non-exploratory (**a**) and exploratory bouts (**b**). (**c**) Same as in (b) for the same cell but after exclusion of outlier head direction points that are illustrated in gray. The right panels show the head-direction tuning curves under the corresponding conditions.

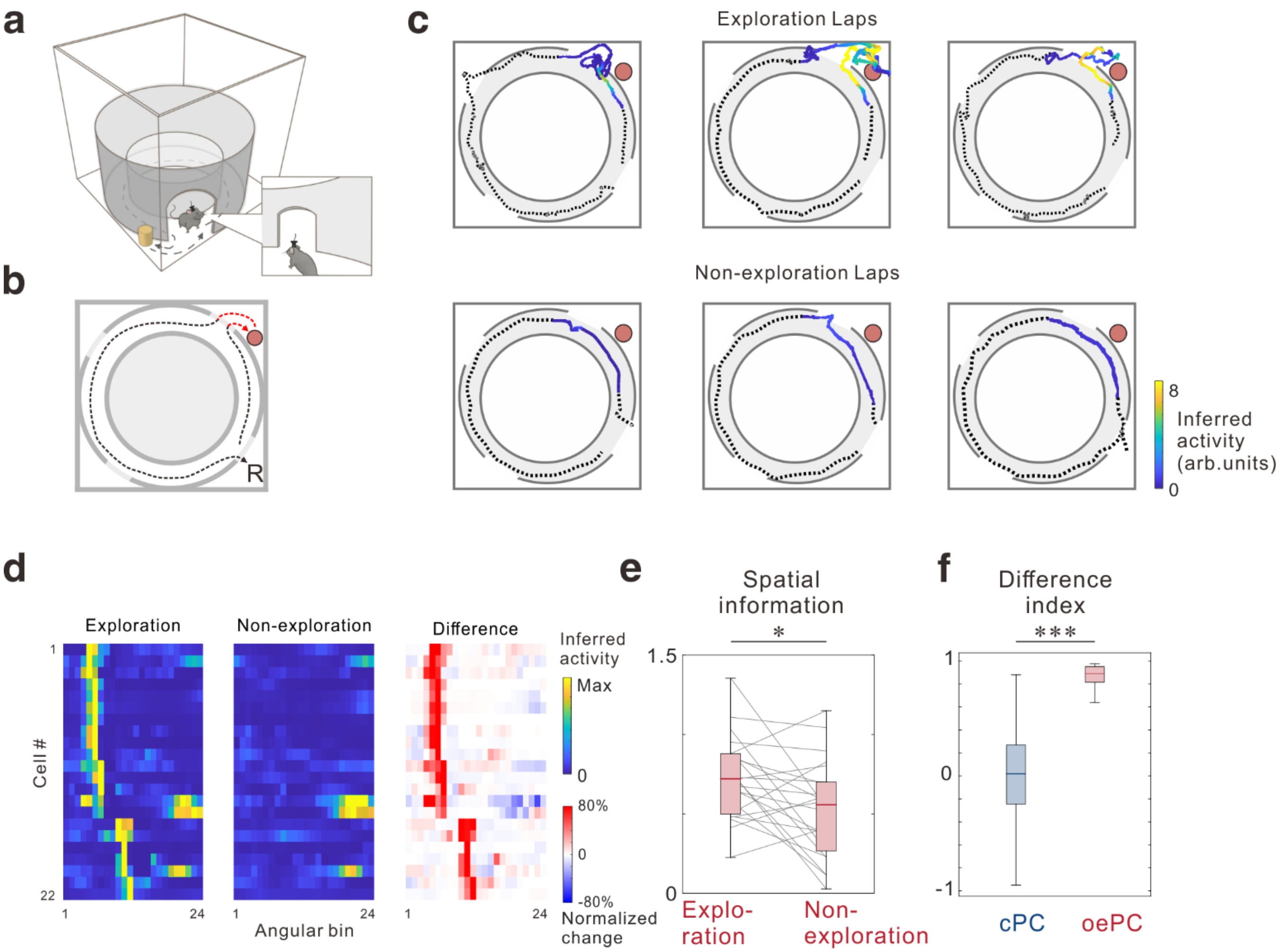

Supplementary Figure 6. Encoding of exploratory intentions by object exploration-dependent place cells (oePCs) for indirectly visible objects. (**a**) The experimental design diagram illustrating the object positioned outside of the track with visibility obstructed by opaque partitions. Following training in this behavioral arena with objects, mice freely explored the concealed object by passing through a designated door or to bypass the door location without engaging in exploration. (**b**) Top view of the maze displaying the location of a sample object and rewards (R). The dashed curves represent the mouse's trajectory during an exploration lap, with the red segment indicating "off-track" positions. (**c**) Color-coded neuronal activity of an example oePCs along the mouse's trajectory, with each panel illustrating a lap during exploration (top) or non-exploration (bottom). These activity maps demonstrate increased oePC activity exclusively during exploration bouts, even when the object's location was well-learned but not directly visible. (**d**) Lap-average activity of oePCs in exploration and non-exploration laps across five mice, sorted by place field centers. Spike rates were normalized to the peak activity of each neuron and sorted according to angular position. The right panel shows difference between the activity map in exploration and non-exploration laps. (**e**) The spatial index during exploratory laps was significantly higher than non-exploratory laps in identified oePCs (n = 22 in 5 mice; P = 0.036; paired Wilcoxon signed rank test). (**f**) The difference index of oePC (red) was significantly higher than that of classic place cells (cPC) (blue, n = 310). P = 1.08×10^{-14}; two-sided Mann-Whitney U test. Box plots show the median (horizontal line), 25–75% range (box) and outliers (whiskers). *** for P < 0.001, ** for P < 0.01, and * for P < 0.05, and NS for no significance. Data and statistical analyses are reported in the Source Data file.

a

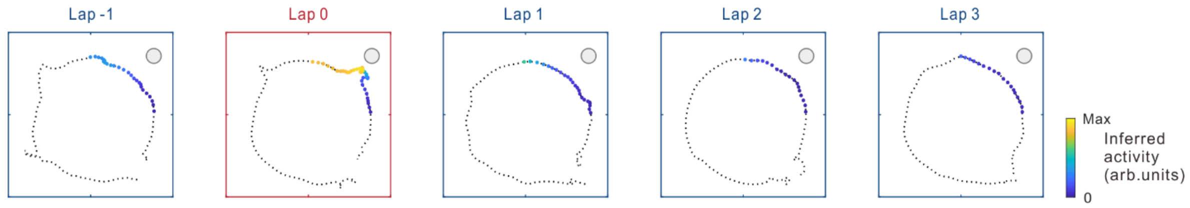

Example oePC 1

b

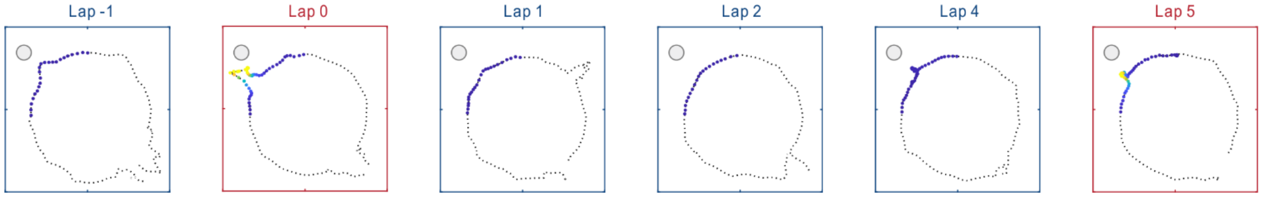

Example oePC 2

c

$$\theta_{pre}^{m} = C_{jm} - (0.5 + m/N)C_{potentiation} \quad (1)$$

$$\theta_{post}^{m} = C_{jm} - C_{tol}\left(C_{jm} - \max_{b \in B} C_{jb}\right) \quad (2)$$

$$\max_{b \in B} C_{jb} < \theta_{pre}^{m} \quad (3)$$

$$C_{jf} \geq \theta_{post}^{m}, \ f \in F \quad (4)$$

Supplementary Figure 7. The activity of object exploration-dependent place cells (oePCs) in laps immediately before and after exploration. (**a-b**) Color-coded activity map of two example oePCs along the mouse's trajectory. Each panel illustrates the laps before and after "Lap 0", which represents the tested exploration lap. These activity maps show that oePCs exhibit increased activity only during exploration bouts (red), but not during subsequent non-exploration laps (blue). (**c**) Formulas are adapted from Monaco *et al.* (2014) to identify possible place-field potentiation events, where θ_{pre}^{m} and θ_{post}^{m} are the baseline maximum threshold and the follow-up minimum threshold, respectively, m is the tested lap, N is the total lap number (1), C_{jm} is the normalized activity of the tested lap (2), C_{jb} is the baseline lap activity (3), C_{jf} is the follow-up lap activity (4), $C_{potentiation}$ = 50% is the nominal amount of relative potentiation, and C_{tol} = 30% is the a tolerance parameter. We excluded the first and last laps in a recording session and identified 101 exploration laps for analysis, among which 16 laps were excluded. A lap with exploration event is considered the place-field potentiation if the formula (3) and (4) are satisfied. We found no events that met the criteria for place-field potentiation among 527 cells × laps in 107 oePCs.

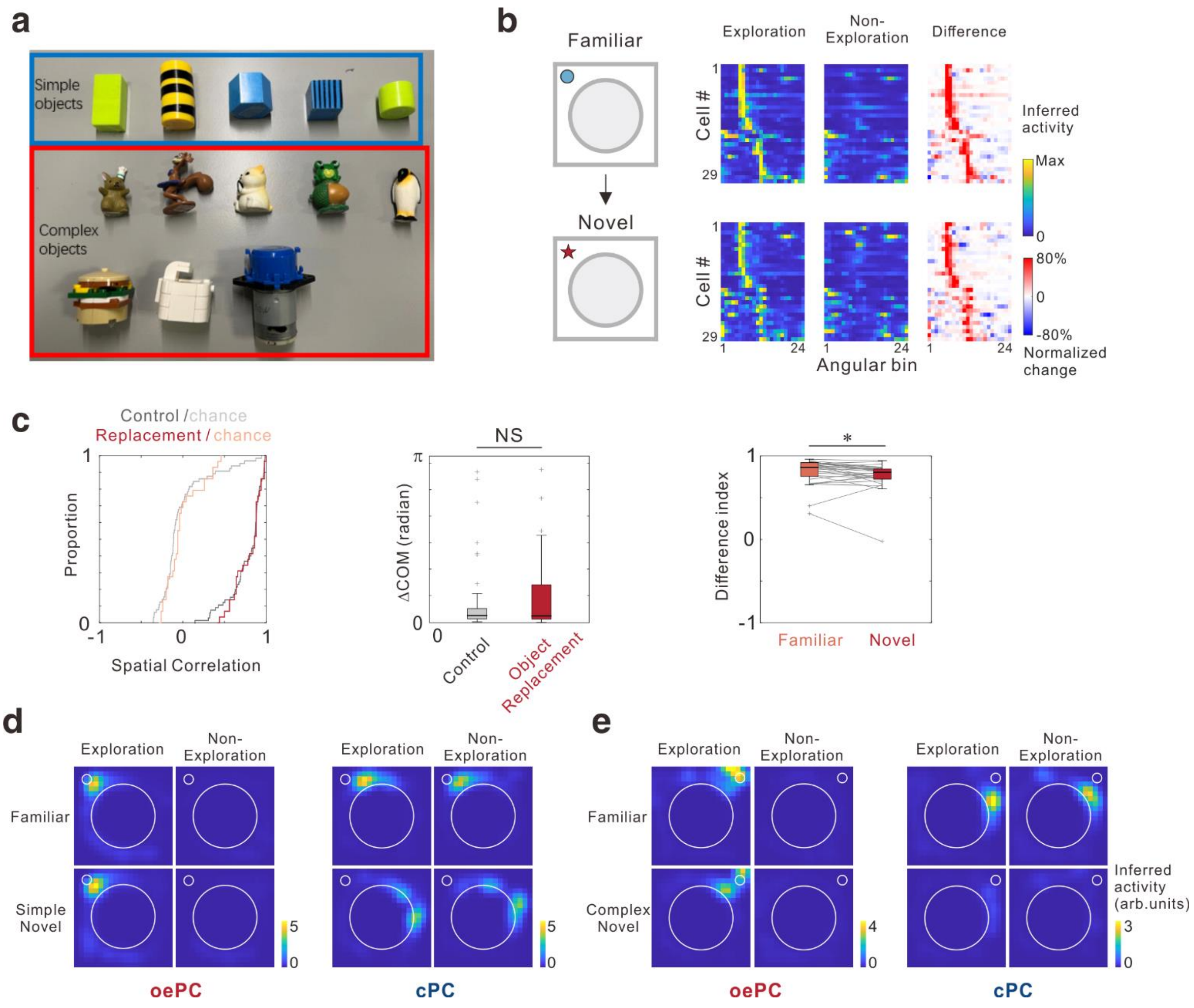

Supplementary Figure 8. Effect of replacing familiar objects with novel complex objects. (**a**) A photo of representative objects used in the experiments. (**b**) Lap-average activity of oePCs during exploration and non-exploration laps, and activity difference upon replacing the familiar object with a novel complex object at the same location. Inferred activity was normalized to the peak activity of each neuron and sorted by place field centers according to angular position across mice. (**c**) Changes in spatial properties of object exploration-dependent place cells (oePCs) during object replacement (red): spatial correlation (P= 0.475; two-sample Kolmogorov-Smirnov test compared to control), ΔCOM (P = 0.971; two-sided Mann-Whitney U test), and the difference index (P = 0.0225; Paired Wilcoxon signed rank test, n = 29 in 4 mice). However, the DI values for both familiar (orange) and novel objects (red) are significantly higher than 0 ($P = 2.56 \times 10^{-6}$ and $P = 2.85 \times 10^{-6}$, respectively; Wilcoxon signed rank test). Box plots show the median (horizontal line), 25–75% range (box) and outliers (whiskers). *** for P < 0.001, ** for P < 0.01, and * for P < 0.05, and NS for no significance. Data and statistical analyses are reported in the Source Data file. (**d**) Substantial alterations were observed in the activity maps of a representative classical place cells (cPC, right), whereas the activity of a representative oePC (left) from the same experiment remained consistent during object replacement. (**e**) Similar to (d), but for the replacement of a complex object.

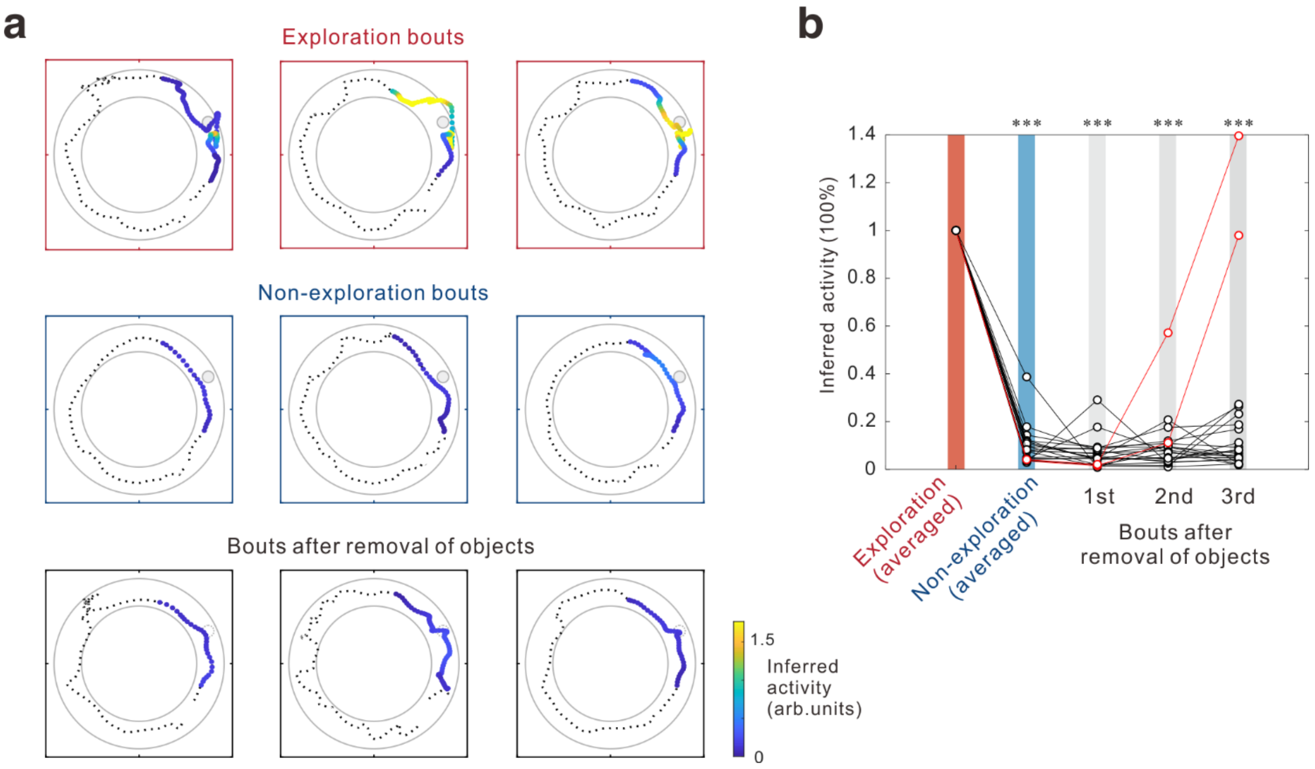

Supplementary Figure 9. The activity of object exploration-dependent place cells (oePCs) before and after object removal. (**A**) Color-coded activity rate of an example oePC along the mouse's trajectory during the exploration/non-exploration bouts when the object occupied a specific location, and during the first three laps after the object was removed. (**B**) Pooled data show that, after the removal of the object, neuronal activity in 20 out of 22 oePCs (black) were similar to those observed during non-exploration bouts, whereas 2 out of 22 oePCs (red lines) exhibited increased activity similar to the property of misplace cells. Inferred activity was averaged for exploration or non-exploration bouts, and shown for the first three laps after the removal of objects. All neural activity was normalized to the mean activity during exploration in each cell (n = 6 mice). Statistical significance was determined by Kruskal-Wallis H tests. *** for $P < 0.001$ compared to the inferred activity during exploration laps. Data and statistical analyses are reported in the Source Data file.

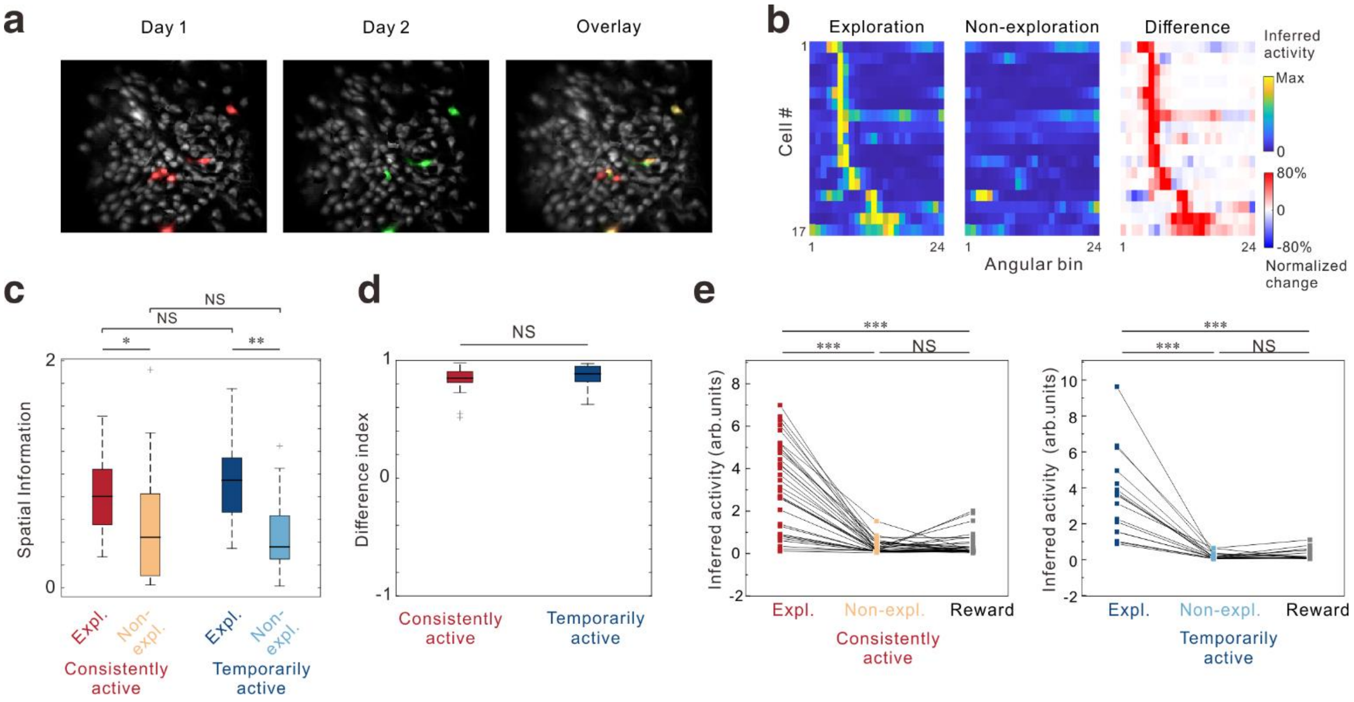

Supplementary Figure 10. Comparisons between consistently and temporarily active object exploration-dependent place cells (oePCs). (**a**) Aligned SPF maps overlaid from two representative recording sessions with one day apart. Yellow cells in the right panel indicate consistently active oePCs that can be tracked across both days, while red cells represent temporarily active oePCs observed on the first day. In all paired cross-day recordings, 60.0% of total cells remained active in recording sessions on subsequent days, while 67.7% of oePCs remained active. (**b**) Lap-average activity of oePCs in exploration (Expl.) and non-exploration (Non-expl.) laps across all mice, sorted by place field centers. Neuronal activity was normalized to the maximum activity of each neuron and sorted according to angular position. The right panel shows difference between the activity map in exploration and non-exploration laps. (**c**) For temporarily active oePCs, spatial information during exploration laps was significantly higher than during non-exploration laps ($n = 17$ cells, 8 mice). However, spatial information during each behavior type was indistinguishable between consistently (red) and temporarily active oePCs (blue; $P = 0.81$, Kruskal-Wallis H test followed by multiple comparison test). (**d**) No significant difference was observed in the difference index between consistently ($n = 34$) and temporarily active oePCs ($P = 0.396$; two-sided Mann-Whitney U test. Box plots show the median (horizontal line), 25–75% range (box) and outliers (whiskers). (**e**) Both consistently and temporarily active oePCs show significantly lower activity during reward periods compared to during object exploration periods, as determined by Kruskal-Wallis H tests. *** for $P < 0.001$, ** for $P < 0.01$, and * for $P < 0.05$, and NS for no significance. Data and statistical analyses are reported in the Source Data file.

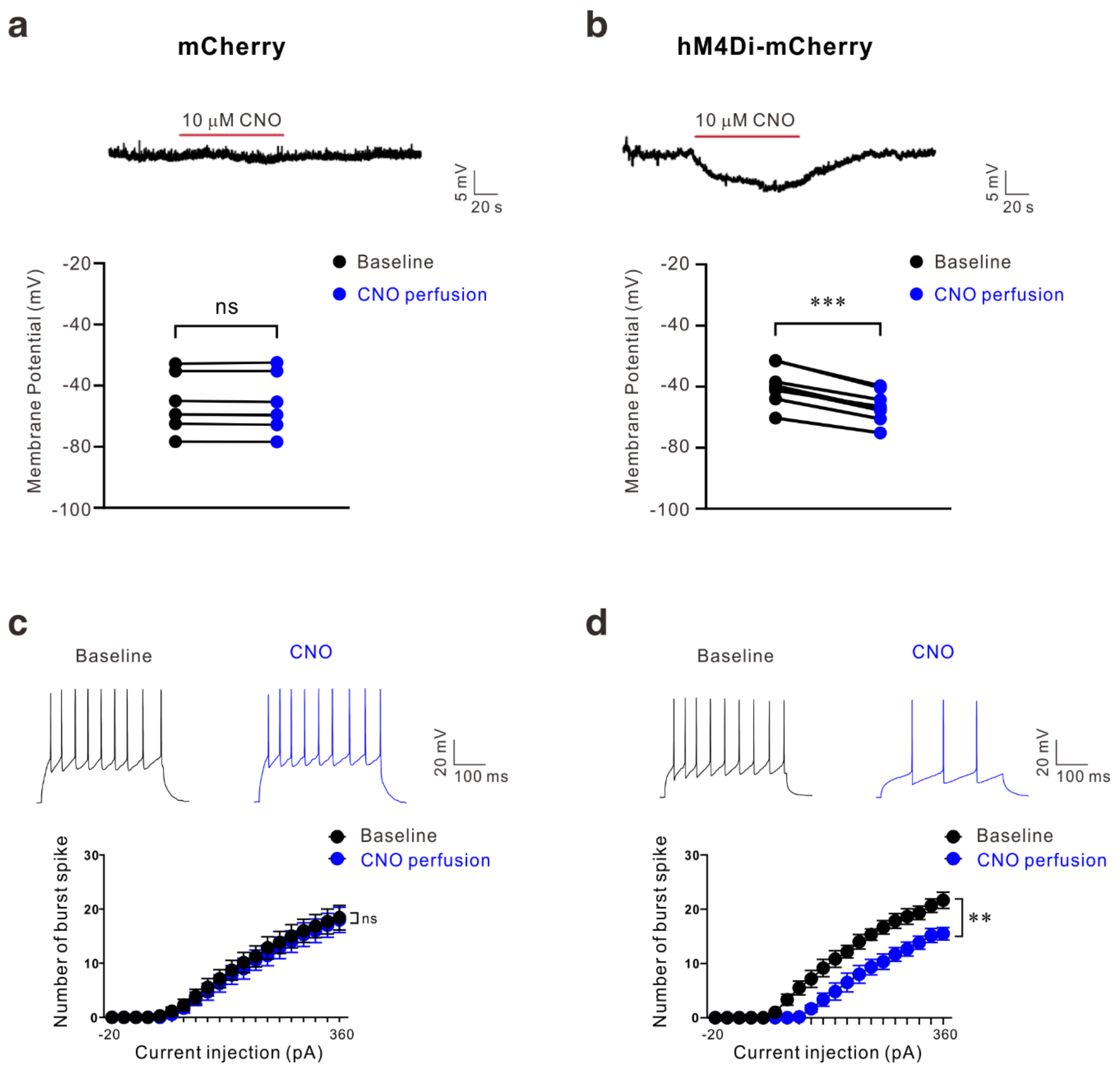

Supplementary Figure 11. Chemogenetic inhibition of neuronal excitability in lateral entorhinal cortex (LEC) neurons. (**a**) Top: Representative trace illustrating changes in membrane potentials recorded from a mCherry-expressing LEC neuron. Bottom: The mean membrane potentials were not significantly affected by application of 10 μM of clozapine-N-oxide (CNO) (n = 7, blue). (**b**) The same as in (a) but for hM4Di-expressing neurons. Application of CNO significantly hyperpolarized the membrane potentials in LEC neurons (n = 8). *** $P < 0.001$ determined by a two-tailed paired t-test. (**c**) Top: Representative traces showing spikes induced by current injection (160 pA) in a mCherry-expressing neuron. Bottom: Application of 10 μM of CNO did not significantly affect number of spikes in response to current steps in mCherry-expressing neurons (n = 7). (**d**) The same as in (c) but for hM4Di-expressing neurons. Application of 10 μM of CNO significantly reduced number of spikes in response to current steps in hM4Di-expressing neurons (n = 6). *** for $P < 0.001$, ** for $P < 0.01$, and * for $P < 0.05$, and ns for no significance as determined by a two-way ANOVA. Data and statistical analyses are reported in the Source Data file.

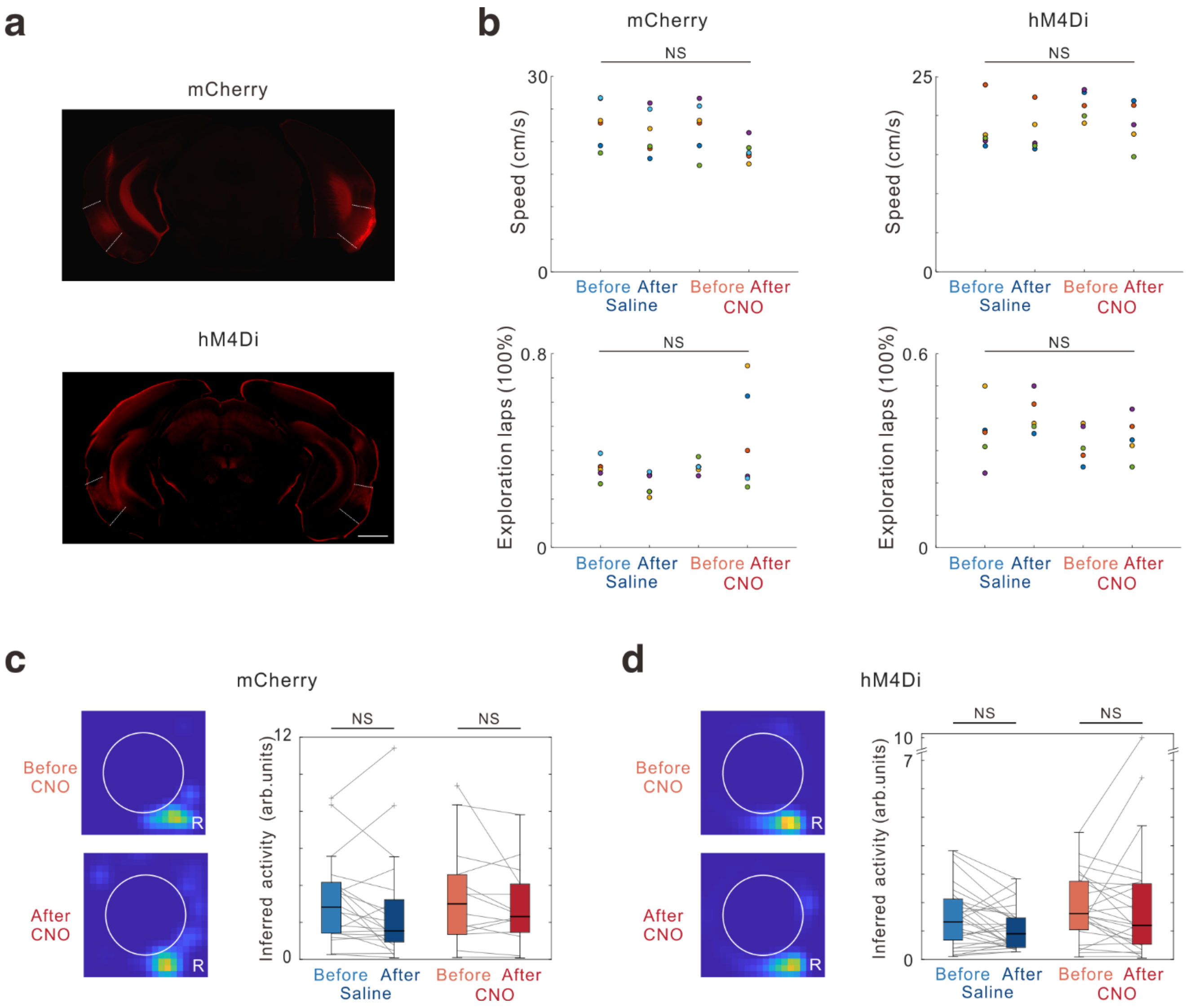

Supplementary Figure 12. Effects of CNO on animal behaviors and reward-associated (RA) cells in mCherry and hM4Di expressing mice. (**a**) Representative images showing hSyn-mCherry or hSyn-hM4Di-mCherry expression in lateral entorhinal cortex (LEC). Scale, 1 mm. (**b**) Application of clozapine-N-oxide (CNO) did not affect the mean running speed (top) or percentage of exploration behaviors (bottom) in mCherry (n = 6 mice) or hM4Di mice (n = 5 mice). NS, no significance as determined by Kruskal-Wallis H tests. (**c**) Application of CNO did not significantly affect activity of reward-associated cells in mCherry-expressing mice. Left: Activity maps of a representative cell that responds to reward site (indicated by "R") before and after CNO treatment. Right: Inferred activity of reward-related cells after treatments of saline (n = 21 cells, blue) or CNO (n = 14 cells, red). (**d**) The same as in (c) but for hM4Di-expressing mice. NS, no significance as determined by paried Wilcoxon signed rank tests. Box plots show the median (horizontal line), 25–75% range (box) and outliers (whiskers). NS, no significance. Data and statistical analyses are reported in the Source Data file.

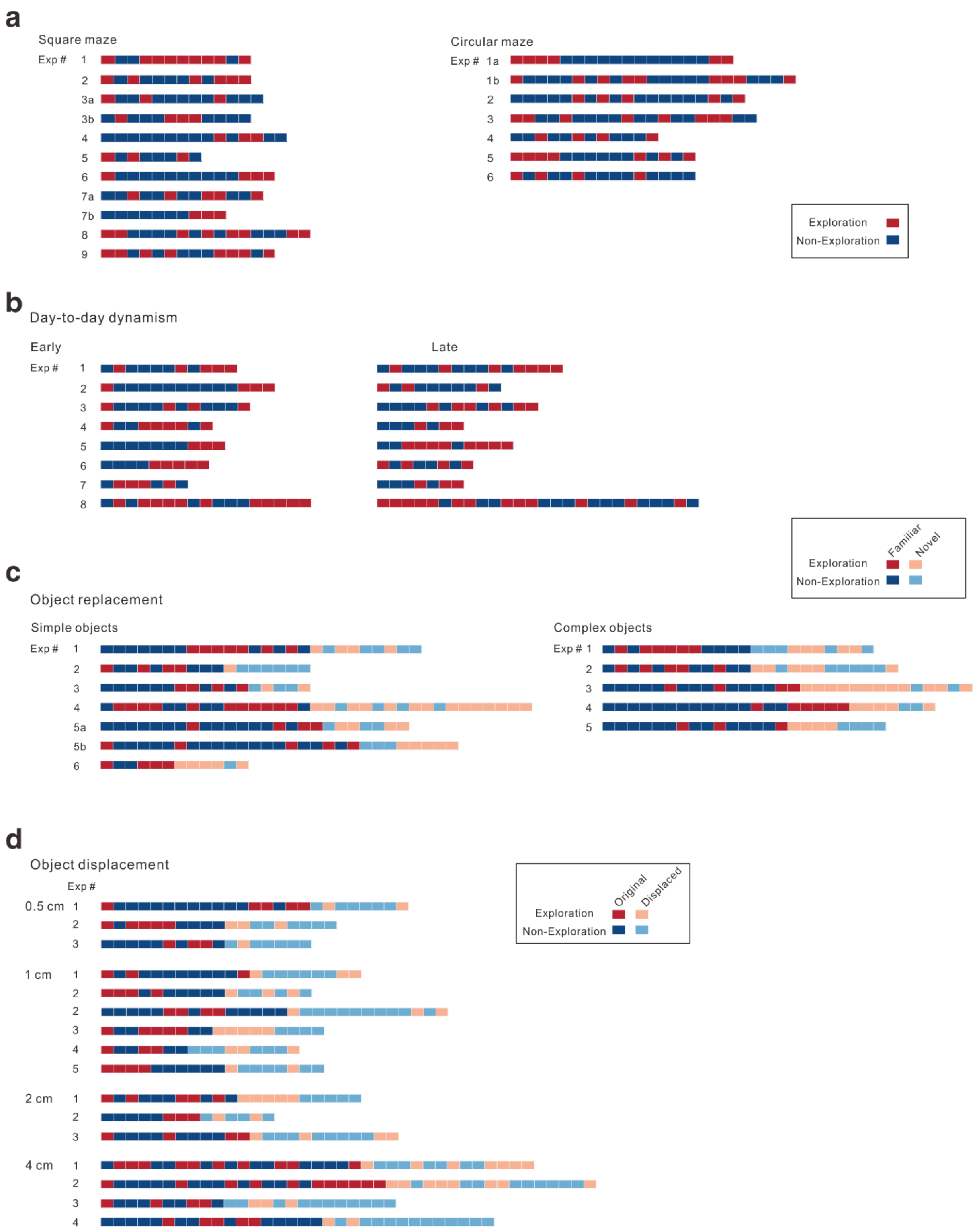

Supplementary Figure 13. The sequencing of laps. (**a**) Identification of oePCs related to Fig. 2. (**b**) Assessment of cross-day activity in oePCs related to Fig. 6. (**c**) Object replacement experiments related to Fig. 5 and Supplementary Fig. 7. (**d**) Object displacement experiments related to Fig. 5.
