# Supplementary material for: Conjunctive encoding of exploratory intentions and spatial information in the hippocampus
Source: Nat Commun. 2024 Apr 15;15:3221. doi: 10.1038/s41467-024-47570-4 (PMC11018604; doi:10.1038/s41467-024-47570-4)
Supplement: Supplementary file 3 — Reporting Summary [file 41467_2024_47570_MOESM3_ESM.pdf]

nature portfolio

Corresponding author(s): Ning Zhou

Last updated by author(s): Feb 28, 2024

Reporting Summary

Nature Portfolio wishes to improve the reproducibility of the work that we publish. This form provides structure for consistency and transparency in reporting. For further information on Nature Portfolio policies, see our Editorial Policies and the Editorial Policy Checklist.

Statistics

For all statistical analyses, confirm that the following items are present in the figure legend, table legend, main text, or Methods section.

n/a	Confirmed	
☐	☒	The exact sample size (*n*) for each experimental group/condition, given as a discrete number and unit of measurement
☐	☒	A statement on whether measurements were taken from distinct samples or whether the same sample was measured repeatedly
☐	☒	The statistical test(s) used AND whether they are one- or two-sided<br>*Only common tests should be described solely by name; describe more complex techniques in the Methods section.*
☒	☐	A description of all covariates tested
☐	☒	A description of any assumptions or corrections, such as tests of normality and adjustment for multiple comparisons
☐	☒	A full description of the statistical parameters including central tendency (e.g. means) or other basic estimates (e.g. regression coefficient) AND variation (e.g. standard deviation) or associated estimates of uncertainty (e.g. confidence intervals)
☐	☒	For null hypothesis testing, the test statistic (e.g. F, t, r) with confidence intervals, effect sizes, degrees of freedom and P value noted<br>*Give P values as exact values whenever suitable.*
☒	☐	For Bayesian analysis, information on the choice of priors and Markov chain Monte Carlo settings
☒	☐	For hierarchical and complex designs, identification of the appropriate level for tests and full reporting of outcomes
☒	☐	Estimates of effect sizes (e.g. Cohen's d, Pearson's r), indicating how they were calculated

Our web collection on statistics for biologists contains articles on many of the points above.

Software and code

Policy information about availability of computer code

Data collection	Neural Ca2+ imaging data and animal's behavior was acquired using Miniscope Controller software available from www.miniscope.org.
Data analysis	The position of the animal was captured simultaneously with Ca2+ imaging using an overhead behavioral camera (30 frames per second) with the MiniScopeControl program https://github.com/daharoni/Miniscope_DAQ_Software. The position and speed of the animal was extracted from the behavior videos using the MiniscopeAnalysis package from https://github.com/daharoni/Miniscope_Analysis. The Ca2+ imaging data was subjected to motion correction using a non-rigid motion correction algorithm (NoRMCorre, available at https://github.com/flatironinstitute/NoRMCorre), the constrained nonnegative matrix factorization for microendoscope data algorithm (CNMF-E, available at https://github.com/zhoupc/CNMF_E), and the algorithm for calibrated spike inference of Ca2+ data using deep networks (CASCADE, available at https://github.com/HelmchenLabSoftware/Cascade). All data was analyzed with custom code in Matlab 2022b.

For manuscripts utilizing custom algorithms or software that are central to the research but not yet described in published literature, software must be made available to editors and reviewers. We strongly encourage code deposition in a community repository (e.g. GitHub). See the Nature Portfolio guidelines for submitting code & software for further information.

Data

Policy information about availability of data

All manuscripts must include a data availability statement. This statement should provide the following information, where applicable:

- Accession codes, unique identifiers, or web links for publicly available datasets
- A description of any restrictions on data availability
- For clinical datasets or third party data, please ensure that the statement adheres to our policy

Data generated in this study are provided in the Source Data file.

Research involving human participants, their data, or biological material

Policy information about studies with human participants or human data. See also policy information about sex, gender (identity/presentation), and sexual orientation and race, ethnicity and racism.

Reporting on sex and gender	N/A
Reporting on race, ethnicity, or other socially relevant groupings	N/A
Population characteristics	N/A
Recruitment	N/A
Ethics oversight	N/A

Note that full information on the approval of the study protocol must also be provided in the manuscript.

Field-specific reporting

Please select the one below that is the best fit for your research. If you are not sure, read the appropriate sections before making your selection.

☒ Life sciences ☐ Behavioural & social sciences ☐ Ecological, evolutionary & environmental sciences

For a reference copy of the document with all sections, see nature.com/documents/nr-reporting-summary-flat.pdf

Life sciences study design

All studies must disclose on these points even when the disclosure is negative.

Sample size	No statistical methods were used to predetermine sample sizes. Sample sizes of mice and cells are similar to other contemporary studies in the field (Gauthier and Tank, Neuron, 2018; GoodSmith et al., Current Biology, 2022; Xu et al., Neuron, 2023).
Data exclusions	Animals with poor Ca2+ imaging quality were not used in behavior experiments. The inclusion criteria for the test sessions were based on sufficient behavioral coverage (see Methods). Exclusion criteria were not predetermined.
Replication	The main findings (Fig. 2-4, Supplementary Fig 1, 2, 5, 7) of the study were replicated in multiple mouse cohorts (total of 15 mice), using two different behavioral apparatuses and various 3D objects (see Supplementary Fig. 2 and Supplementary Fig. 8a for raw neuronal images in each animal and object pictures). Replication of individual findings (Fig. 1, 5-7 and Supplementary Fig. 3, 4, 6, 8-12) involved 3-8 mice each, as detailed in the corresponding figure legends.
Randomization	Naive animals were randomly assigned to either the hM4Di or control groups. Animals were not assigned to groups in other experiments.
Blinding	The major experiments did not involve administering treatments/drugs to animals; the CNO experiments compared pre- and post-treatment results within each animal. As no treatment comparisons between animal groups were involved, investigators were not blinded to animal identities during data collection. Nonetheless, trained individuals independently and blindly annotated exploration and non-exploration behaviors for data analysis.

Reporting for specific materials, systems and methods

We require information from authors about some types of materials, experimental systems and methods used in many studies. Here, indicate whether each material, system or method listed is relevant to your study. If you are not sure if a list item applies to your research, read the appropriate section before selecting a response.

Materials & experimental systems

n/a	Involved in the study
☒	☐ Antibodies
☒	☐ Eukaryotic cell lines
☒	☐ Palaeontology and archaeology
☐	☒ Animals and other organisms
☒	☐ Clinical data
☒	☐ Dual use research of concern
☒	☐ Plants

Methods

n/a	Involved in the study
☒	☐ ChIP-seq
☒	☐ Flow cytometry
☒	☐ MRI-based neuroimaging

Animals and other research organisms

Policy information about studies involving animals; ARRIVE guidelines recommended for reporting animal research, and Sex and Gender in Research

Laboratory animals	Male C57BL/6J mice aged 8-16 weeks were used in the study. All mice were individually housed under a 12-hour light/dark cycle with ad libitum access to water and maintained at a constant temperature (20-26 °C) and 40-60% humidity.
Wild animals	The study did not involve wild animals.
Reporting on sex	Only male mice were used.
Field-collected samples	The study did not involve field-collected samples.
Ethics oversight	The animal experiments reported in this study were conducted in accordance with the guidelines for the Care and Use of Experimental Animals at ShanghaiTech University, which were approved by the Institutional Animal Care and Use Committee.

Note that full information on the approval of the study protocol must also be provided in the manuscript.

Plants

Seed stocks	N/A
Novel plant genotypes	N/A
Authentication	N/A
